# Supplementary material for: DNA Damage Induction Alters the Expression of Ubiquitin and SUMO Regulators in Preimplantation Stage Pig Embryos
Source: Int J Mol Sci. 2022 Aug 25;23(17):9610. doi: 10.3390/ijms23179610 (PMC9455980; doi:10.3390/ijms23179610)
Supplement: Supplementary file 1 [file ijms-23-09610-s001.zip › ijms-1862573-supplementary.pdf]

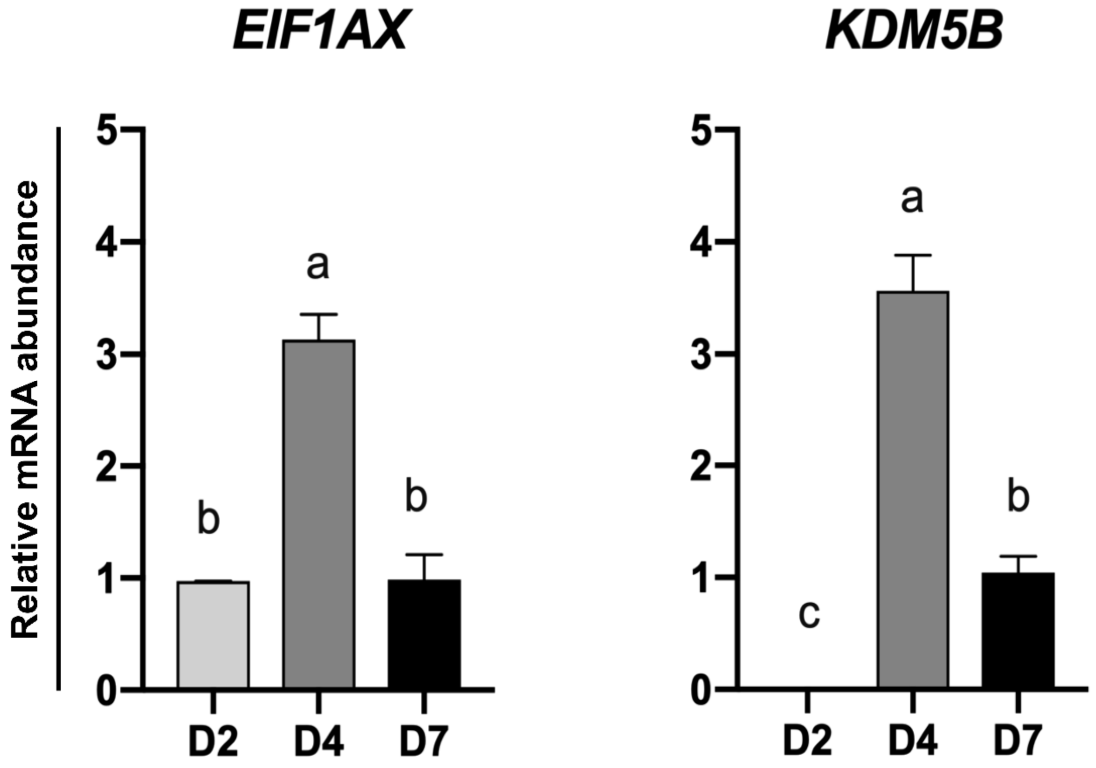

Supplementary Figure S1. Relative mRNA abundance of *EIF1AX* and *KDM5B* genes, which are known to be upregulated during the major-EGA stage in pig embryos. Different letters indicate statistical differences (P<0.05) between developmental stages. These results confirm the proper developmental stages of embryos used in this study with regards to the EGA.
